# Pulmonary Histoplasmosis in People Living with Human Immunodeficiency Virus in French Guiana: Clinical Epidemiology, Medical Imaging and Prognostic

**DOI:** 10.1007/s11046-023-00799-x

**Published:** 2023-10-15

**Authors:** Morgane Bourne-Watrin, Antoine Adenis, Gary Doppelt, Magaly Zappa, Loïc Epelboin, Mathieu Nacher, Jeanne Bigot, Kinan Drak Alsibai, Romain Blaizot, Denis Blanchet, Magalie Demar, Geneviève Guillot, Félix Djossou, Pierre Couppié

**Affiliations:** 1 Service de Dermatologie-Vénérologie, Centre Hospitalier de Cayenne, Cayenne, France; 2 Centre d’Investigation Clinique Antilles Guyane, Inserm CIC 1424, Centre Hospitalier de Cayenne, Cayenne, France; 3 Service de Radiologie, Centre Hospitalier de Cayenne, Cayenne, France; 4 Service de Parasitologie-Mycologie, Centre Hospitalier Saint Antoine APHP, Paris, France; 5 Laboratoire d’Anatomie et Cytologie Pathologique, Centre Hospitalier de Cayenne, Cayenne, France; 6 Laboratoire de Parasitologie-Mycologie, Centre Hospitalier de Cayenne, Cayenne, France; 7 Unité des Maladies Infectieuses et Tropicales, Centre Hospitalier de Cayenne, Cayenne, France; 8 Service de Médecine B, Centre Hospitalier de Cayenne, Cayenne, France

**Keywords:** Pulmonary histoplasmosis, HIV, Miliary, Nodules, French Guiana

## Abstract

**Background:**

Histoplasmosis is mainly described as a disseminated disease in people living with HIV (PLHIV). Compared to historical descriptions in immunocompetent individuals, knowledge is lacking on the detailed clinical and radiological findings and outcomes of pulmonary histoplasmosis (PH). Overlooked or misdiagnosed with other AIDS-defining condition, prognostic of PLHIV may be at risk because of inappropriate care.

**Methods:**

A retrospective multicentric study was conducted in PLHIV from French Guiana between January 1988 and October 2019. Proven PH were documented through mycological direct examination, culture, or histology. Patients with concomitant respiratory infections were excluded.

**Results:**

Among 65 patients, sex ratio M:F was 2.4 with a median age of 39 years [IQR 25–75%: 34–44]. Median CD4 count was 24 cells/mm^3^ [11–71], with histoplasmosis as the AIDS-defining condition in 88% and concomitant AIDS-defining conditions in 29%. Clinical findings were fever (89%), cough (58%), dyspnea (35%), expectoration (14%), and hemoptysis (5%). Sixty-one X-rays and 24 CT-scans were performed. On X-rays, an interstitial lung disease was mainly found (77%). On CT-scans, a nodular pattern was predominant (83%): mostly miliary disease (63%), but also excavated nodules (35%). Consolidations were present in 46%, associated with miliary disease in 21%. Thoracic lymphadenopathies were found in 58%, mainly hilar and symmetric (33%). Despite antifungal treatment, case-fatality rate at one month was 22%.

**Conclusion:**

When faced with an interstitial lung disease on X-rays or a miliary pattern on CT-scans in advanced PLHIV, physicians in endemic areas, apart from tuberculosis or pneumocystosis, should include histoplasmosis as part of their differential diagnoses.

**Supplementary Information:**

The online version contains supplementary material available at 10.1007/s11046-023-00799-x.

## Introduction

*Histoplasma capsulatum* var *capsulatum *(*H. capsulatum*) is the most frequent endemic mycosis in patients living with the acquired immunodeficiency syndrome (AIDS) [1]. Unlike in immunocompetent patients, it presents itself as a disseminated form in 95% of cases [2] and is mostly fatal without appropriate antifungal treatment [1].

In French Guiana, despite highly active antiretroviral therapy (HAART), disseminated histoplasmosis (DH) remains the most common AIDS-defining illness with an incidence of 15.4 per 1000 person–years HIV infected [3] and used to be the first cause of AIDS related death [4]. In endemic areas, death rates caused by HIV-associated histoplasmosis differ between the USA (12–23%) and South America (19–39%) [5]. These differences could be due to diagnostic delays related to the lack of diagnostic facilities and non-invasive diagnostic methods outside the USA [6]. It could also be related to the fact that histoplasmosis may be misdiagnosed as “resistant not documented tuberculosis” and thus mistreated, leading to death [4, 7].

A modelling study in people living with human immunodeficiency virus (PLHIV) has compared the burden of histoplasmosis to tuberculosis and estimated that in 2012, 671–9394 deaths could be attributed to histoplasmosis while 5062 were caused by tuberculosis. Central America, northernmost part of South America and Argentina were defined as hotspot areas [8]. Numerous people in Latin America are dying from this neglected curable disease mainly because of diagnostic delays when the prognosis in these immunosuppressed patients relies on the rapid initiation of antifungal therapy [3].

In Latin America, when a patient with uncontrolled HIV infection presents respiratory symptoms, the diagnosis of histoplasmosis is overlooked, overtaken by the diagnoses of tuberculosis or *Pneumocystis jirovecii* pneumonia (PJP) when an interstitial lung disease is seen on chest radiograph. This leads to even further inappropriate presumptive therapies when rapid noninvasive tests to identify histoplasmosis are not available.

This lack of knowledge on the radiological findings of the pulmonary focalization of histoplasmosis in PLHIV could be due to the fact that it is not well described in the literature, and that the presentation differs from that of immunocompetent patients [9].

Thus, the aim of our study was to describe the clinical, biological and radiological presentation of the pulmonary focalization of histoplasmosis in PLHIV in French Guiana. A secondary objective was to evaluate the prevalence of pulmonary histoplasmosis (PH) among all histoplasmosis cases in PLHIV.

## Methods

### Study Design and Populations

The study was a cross-sectional retrospective, descriptive, multicentric study between January 1st, 1988 and October 1st, 2019.

The target population was all known PLHIV followed in one of the three hospitals in French Guiana, Cayenne, Kourou and Saint-Laurent-du-Maroni. The source population was patients with an episode of HIV-associated histoplasmosis included in the French Hospital database on HIV (FHDH) and its subsequent histoplasmosis and HIV database of French Guiana.

The inclusion criteria were: age > 18 years; confirmed HIV infection using western blot; a proven episode of PH either by direct examination (MDE) and culture in mycology or anatomical pathology and cytology (APC) (excluding immunodiagnosis and PCR) performed on a variety of respiratory samples following EORTC/MSG criteria [10]. Unproven histoplasmosis (successful empirical antifungal therapy), histoplasmosis of another organ with thoracic radiological abnormality but without positive pulmonary sample, or diagnosis of a concomitant respiratory infection were excluded.

### Study Conduct

We screened for all respiratory samples of participants with an episode of HIV-associated histoplasmosis and included in the Histoplasmosis and HIV database of French Guiana. PH was defined as the identification of *H. capsulatum* with either MDE on May-Grünwald Giemsa-stained slides or mycological culture on Sabouraud media or APC examination using Periodic Acid Schiff or Gomori–Grocott stainings. Respiratory samples under study were bronchoalveolar-lavage (BAL), pulmonary biopsy, tracheobronchial suctioning or sputum.

Data were retrospectively and routinely collected on a standardized form and entered in the database. The data collected concerned socio-demographic data (sex, age, country of origin); clinical data (habitus, past medical history, signs and symptoms on admission); laboratory (immunovirological assessment, standard biological examinations, mycology and pathology on any type of samples) and medical imaging findings; antifungal therapy data (duration, dosage, route of administration); survival status 30 days after the start of antifungal treatment (case fatality rate). With regards to the objective of the study we reviewed and detailed pulmonary or mediastinal abnormalities on chest X-rays and CT-scans data using the *Glossary of Terms for Thoracic Imaging* of the Fleischner Society [11]. On chest X-rays, abnormalities were classified in 5 patterns: nodular pattern, interstitial lung disease, consolidation, cavitary pattern, and “others” [12]. Consolidation covered the terms “alveolar pattern”, “alveolar opacification”, “parenchymal opacification”, or “air-space opacity”. Nodular presentation was individualized as a specific pattern, because nodules could be present as diffuse micronodules (miliary disease) and/or as isolated nodules. Micronodules were defined as nodules < 3 mm. The term “infiltrates” was not considered because it could refer to interstitial or parenchymal injuries. CT-scans were blindly reviewed by two radiologists (one junior and one senior) and send to a third-one in a reference center in case of discrepancy.

Induction therapy, maintenance therapy and secondary prophylaxis were defined following the “Aidsinfo Guidelines” for Histoplasmosis in PLHIV [13]. Relapse was defined as a new documented infection after three months of clinical recovery [14]. Severe cases were defined according to the 2020 WHO Guidelines for diagnosing and managing DH among PLHIV criteria including ≥ 1 among: respiratory or circulatory failure, neurological signs, renal failure, coagulation anomalies and alteration of the WHO performance status > 2 [15]. Respiratory failure included clinical respiratory distress or oxygen blood pressure level < 70 mmHg. Circulatory (mean blood pressure < 70 mmHg), renal failure (creatinine level > 1.70 mg/dL) and coagulation anomalies were defined to correspond to a SOFA score of 1 [16].

### Statistical Analysis

The statistical analysis was performed with Microsoft Excel 2019. Frequencies and proportions were calculated for categorical variables. For quantitative variables, medians were calculated with 25–75% interquartile ranges [IQR 25–75%].

### Ethics Statement

All HIV-infected patients were included in the DMI-2 database, administered by the Regional Coordination for the fight against HIV (COREVIH). This database is included in the FHDH, the national cohort of PLHIV. In French Guiana, socio-demographic, clinical, biological, radiological and therapeutics data have been prospectively included since January 1st, 1992. This database has received approval by the French regulatory authority, the Commission Nationale Informatique et Libertés (CNIL) on November 27, 1991. All included patients signed an informed consent form. Similarly, patients hospitalized between 1988 and 1992 were retrospectively included following their informed consent. As it is described elsewhere, the main objective of the FHDH hospital cohort is to study the evolution, morbidity and mortality of PLHIV [17]. The histoplasmosis and HIV anonymized database was also approved by the CNIL (No. JZU0048856X, 07/16/2010), the French National Institute of Health and Medical Research institutional review board (CEEI INSERM) (IRB0000388, FWA00005831 18/05/2010), and by the Comité Consultatif pour le Traitement de l’Information pour la Recherche en Santé (CCTIRS) (No. 10.175bis, 10/06/2010) [4].

## Results

### Baseline Characteristics

**Fig. 1:**
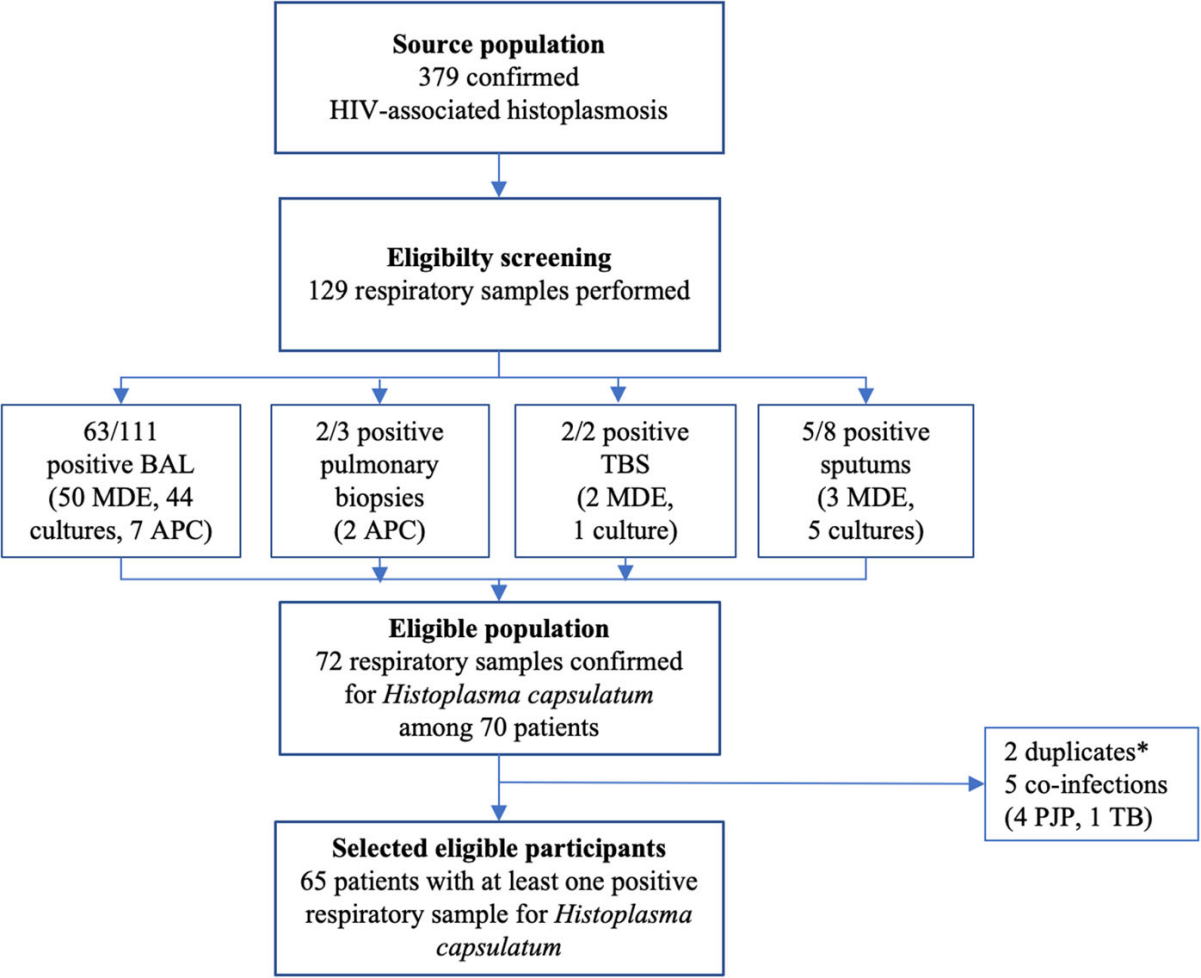
Flow chart of pulmonary histoplasmosis among proven episode of HIV-associated histoplasmosis in French Guiana, 1988–2019. *Patients positive for several respiratory samples at the same time (MDE or culture or APC); *APC* anatomical pathology and cytology, *BAL* broncho alveolar lavage, *MDE* mycological direct examination, *PJP* *Pneumocystis jirovecii* pneumonia, *TB* tuberculosis, *TBS* tracheobronchial suctioning

Among 379 proven episodes of HIV-associated histoplasmosis we included 65 patients with a proven PH, representing a prevalence of 17% (Fig. 1). All cases but one were incident. One case was diagnosed with DH without pulmonary involvement 7 years prior and classified as a relapse. Two cases were diagnosed before 1990, 17 in the 1990’s, 27 in the 2000’s, 19 in the 2010’s.

Participants mainly originated from Haiti and French Guiana (Table 1). Median age was 39 years [34–44] with a sex ratio M:F of 2.4. Seven patients were tobacco smoker. Three patients (5%) were under HAART on admission and none of them was virally suppressed. Histoplasmosis was the AIDS defining condition in 88%, concomitant to another AIDS-defining condition in 29%, the most common events being recurrent HSV mucocutaneous infections (11%) and esophageal candidiasis (9%).

**Table 1:** Baseline data of 65 cases of HIV-associated pulmonary histoplasmosis in French Guiana between 1988 and 2019

	No/total (%) Median [IQR 25–75%]
Sex ratio M:F	2.4
Median age (years) (n = 65)	39 [34–44]
Place of birth	
Haiti	24/65 (37)
French Guiana	20/65 (31)
Suriname	9/65 (14)
Brazil	5/65 (8)
Mainland France	3/65 (5)
Guadeloupe	1/65 (2)
Unknown	3/65 (5)
Median time spent in French Guiana (months) (n = 49)	8.4 [4–14]
Transmission mode of HIV	
Heterosexual	54/65 (83)
Injecting drug user	2/65 (3)
Unknown	9/65 (14)
Drug addiction	10/25 (40)
Tobacco	7/25 (28)
Alcohol	5/25 (20)
Marijuana	3/25 (12)
Crack	3/25 (12)
Cocaine	2/25 (8)
Multiple drug addiction	7/25 (28)
Antiretroviral therapy	3/65 (5)
Median time spent in hospitalization (days) (n = 32)	26.5 [21–36.25]
Median time between diagnosis of HIV and histoplasmosis (years) (n = 46)	1 [0–4]
Histoplasmosis as AIDS-defining condition	57/65 (88)
Previous AIDS-defining conditions*	8/65 (12)
Tuberculosis	3/65 (5)
Recurrent mucocutaneous infections due to HSV	3/65 (5)
Esophageal candidiasis	2/65 (3)
*Pneumocystis jirovecii* pneumonia	1/65 (2)
Cerebral toxoplasmosis	1/65 (2)
*Salmonella* sp. bacteremia	1/65 (2)
Wasting syndrome due to HIV	1/65 (2)
Recurrent bacterial pneumonia	1/65 (2)
Concomitant AIDS-defining conditions^†^	19/65 (29)
Recurrent mucocutaneous infections due to HSV	7/65 (11)
Esophageal candidiasis	6/65 (9)
CMV disease	2/65 (3)
Chronic intestinal isosporiasis	2/65 (3)
Extrapulmonary *Mycobacterium avium complex* infection	1/65 (2)
Cerebral toxoplasmosis	1/65 (2)
*Salmonella* sp. bacteremia	1/65 (2)

*AIDS* acquired immunodeficiency syndrome, *CMV* cytomegalovirus, *HSV* herpes simplex virus

n = number of available data; medians are presented with 25–75% interquartile ranges in brackets

*Multiple associated infections possible; ^†^as the wasting syndrome could be secondary to histoplasmosis, we did not consider this syndrome as an associated AIDS-defining condition. Concomitant tuberculosis or pneumocystosis were exclusion criteria

### Clinical and Laboratory Findings

Medical examination upon admission found fever in 89% and an impaired WHO performance status score in 43% of patients. Median duration of fever was 21 days [10–30].

Pulmonary symptoms were present in 80%: cough (58%), dyspnea (35%), sputum (14%) and hemoptysis (5%). No chest pain nor signs of pericarditis were described. Superficial lymphadenopathies, splenomegaly and hepatomegaly were found in 11%, 18% and 25% respectively (Table 2).

**Table 2:** Clinical examination and laboratory findings upon admission of pulmonary HIV-associated histoplasmosis cases

	No/total (%) Median [IQR 25–75%]
*Clinical examination*	
General state	
Impaired WHO general performance score > 2	28/65 (43)
Weight loss	19/22 (86)
Systolic arterial pressure ≤ 90 mmHg	3/26 (12)
Fever (> 38.3 °C)*	58/65 (89)
Median duration of fever (days) (n = 40)	21 [10–30]
Superficial lymphadenopathy^†^	7/65 (11)
Size [2–5 cm]	7/65 (11)
Size > 5 cm	0/65
Splenomegaly	12/65 (18)
Splenomegaly and concomitant lymphadenopathy	3/65 (5)
Hepatomegaly	16/65 (25)
Hepatomegaly and concomitant lymphadenopathy	2/65 (3)
Hepatomegaly and concomitant splenomegaly	6/65 (9)
Pulmonary signs and symptoms	52/65 (80)
Chest pain	0/65
Respiratory failure	4/65 (6)
Dyspnea	23/65 (35)
Cough	38/65 (58)
Sputum	9/65 (14)
Hemoptysis	3/65 (5)
Abnormal auscultation	9/15 (60)
Crackles	2/15 (13)
Ronchi	3/15 (20)
Hypoventilation	4/15 (27)
Wheezing	0/15
Cutaneous lesions^‡^	15/65 (23)
Oral lesions^§^	11/65 (17)
Digestive signs and symptoms	42/65 (65)
Abdominal pain	21/65 (32)
Diarrhea^II^	20/65 (31)
Abdominal complication^¶^	4/65 (6)
Neurological signs and symptoms**	9/65 (14)
Ocular signs	0/65
*Laboratory findings*	
CD4 count < 200 cells/mm^3^	57/61 (93)
Median CD4 count (cells/mm^3^) (n = 60^††^)	24 [11–71]
Median HIV viral load (copies/mL) (n = 20)	175 695^‡‡^ [70 665–500 250]
Hemoglobin level < 11.5 g/dL	55/60 (92)
Platelet count < 150,000 cells/μL	29/59 (49)
Neutrophil count < 1500 cells/μL	7/57 (12)
Ferritin level > 1000 mg/L	19/24 (79)
Lactate dehydrogenase level > 600 U/L	22/50 (44)
Median C reactive protein level (mg/L) (n = 51)	74 [42–147]
Creatinine level > 1 mg/dL	17/63 (27)
ASAT level > 34 U/L	42/58 (72)
ALAT level > 55 U/L	10/57 (18)
Median ASAT/ALAT ratio (n = 57)	2.4 [1.9–2.9]
Alkaline phosphatase level > 150 U/L	23/57 (40)
γ-Glutamyl transferase level > 50 U/L	45/56 (80)

*ALAT* alanine aminotransferase, *ASAT* aspartate aminotransferase, *WHO* World Health Organization

n = number of available data; *median temperature: 39 °C [39, 40]; ^†^unilateral or bilateral lymph node’s involvement were considered as a single area’s involvement; ^‡^Papule, ulceration, prurigo, superficial skin mycosis, budding genital injury, zona scar, microcirculatory involvement, macular rash, cutaneous nodule

^§^Ulceration, oral mycosis, ulcerovegetating lesion, vesicle, ulcerous nodule, erosion; II watery diarrhea in 85%, ^¶^2 ascites, 1 perforation, 1 hemorrhage, 1 peritonitis **coma, encephalitis, confusion, peripheral neuropathy, ideomotor downturn, meningeal irritation, cerebellar syndrome, focal deficiency; ^††^one patient was not included since his precise CD4 count was not known, we only knew it was “less than 25 cells/mm^3^”, even so, he is still below the median; ^‡‡^5.2 log

Among 60 patients, the median CD4 count was 24 [11–71] and 93% had a CD4 count < 200 cells/mm^3^ with a range of 1–295 cells/mm^3^. Anemia (hemoglobin level < 11.5 g/dL), thrombocytopenia (platelet count < 150,000 cells/μL) and neutropenia (neutrophil count < 1500 cells/μL) were found in 92%, 49% and 12% respectively. Lactate dehydrogenase level > 600UI/L was found in 44%. The median C-reactive-protein level was 74 mg/L [IQR 42–147].

### Imaging Findings

Chest X-rays were performed in 61 patients (94%) and were abnormal in 53 (87%) with several lung abnormalities in 30% and a diffuse distribution in 89% (Table 3). The main abnormality was an interstitial lung disease in 77%, mainly presenting as a reticular or reticulonodular pattern; micronodules, nodules and thoracic lymphadenopathies were seen in 18%, 8% and 10%, respectively. Two patients (3%) with a normal chest X-ray ultimately had an abnormal CT-scan.

**Table 3:** HIV-associated pulmonary histoplasmosis findings on chest X-ray

	Chest X-rays No/total (%) Median [IQR 25–75%]
*Abnormal imaging*	53/61 (87)
> 1 distinct lung abnormality	18/61 (30)
Median number of lesions when > 1 abnormality (n = 53)	3 [2–3.75]
*Distribution*	
Diffuse distribution	47/53 (89)
Focal distribution	6/53 (11)
Upper lobes	1/53 (2)
Central/mediastinal part	4/53 (8)
Lower lobes	2/53 (4)
Unilateral	5/53 (9)
Bilateral but not diffuse	1/53 (2)
*Chest radiographic findings*	
Interstitial lung disease	47/61 (77)
Intralobular lines	8/61* (13)
Non characterizable abnormality	39/61 (64)
Nodular pattern	15/61 (25)
Micronodules (< 3 mm)	11/61 (18)
Diffuse distribution	10/61 (16)
Focal distribution	1/61 (2)
Nodules (3–30 mm)	5/61 (8)
Multiple and diffuse distribution	3/61 (5)
2–5 and focal distribution	2/61 (3)
Solitary nodule	0/61
Intrathoracic mass	1/61 (2)
Consolidation	9/61 (15)
Cavitary pattern	5/61 (8)
Multiple caverns	2/61 (3)
Unique cavern	3/61(5)
Intrathoracic lymphadenopathy†	6/61 (10)
Atelectasis	1/61 (2)
Pleural effusion	2/61 (3)

*Kerley B lines; ^†^intrathoracic lymphadenopathies were defined as a single abnormality, whether they were mediastinal, hilar, unilateral or bilateral. Axillary and cervical lymph nodes were not described on the thoracic imaging

Chest CT-scan were performed in 24 patients (37%), and were all abnormal (Table 4). Eighteen of the 24 CT-scans could be completely reviewed but 6 could not be found so the analysis was based on the interpretation performed at the time of the examination. Lesions were diffuse in 79% and unilateral in 8% of the cases. The main abnormality was a nodular pattern in 83%, composed of micronodules (63%), all in the form of miliary disease with random distribution, nodules > 3 mm (35%) and unique or multiple excavated nodules (35%). In one patient micronodules presented like fireworks, in the so called “cluster sign”. Consolidations were present in 11 patients (46%), isolated in 4 and associated with miliary disease in 5. Of the four patients with isolated consolidations, one was diffuse and bilateral, one was single and lobar and two were perihilar with ground-glass-opacities presenting as batwings opacities. Intrathoracic lymphadenopathies were reported in 58% of patients, mostly bilateral, hilar and symmetric (57%); one patient presented with necrotic lymphadenopathies.

**Table 4:** HIV-associated pulmonary histoplasmosis findings on thoracic CT-scan

	Thoracic CT-scans No/total (%) Median [IQR 25–75%]
*Abnormal imaging*	24/24 (100)
> 1 distinct lung abnormality	20/24 (83)
Median number of lesions when > 1 abnormality (n = 24)	4 [3–5.25]
*Distribution*	
Diffuse distribution	19/24 (79)
Focal distribution	5/24 (21)
Upper lobes	0/24
Central/mediastinal part	3/24 (13)
Lower lobes	2/24 (8)
Unilateral	2/24 (8)
Bilateral but not diffuse	3/24 (13)
*CT-scan findings*	
Nodular pattern	20/24 (83)
Micronodules (< 3 mm)	15/24 (63)
Miliary/hematogenous spread	15/24 (63)*
Nodules (3–30 mm)	7/20^†^ (35)
Multiple and diffuse distribution	1/20^†^ (5)
2–5 and focal distribution	6/20^†^ (30)
Solitary nodule	0/20^†^
Association of micronodules and nodules	2/20^†^ (10)
Excavated nodules	7/20^†^ (35)
Micronodules	5/20^†^ (25)
Nodules	2/20^†^ (10)
Multiple and diffuse distribution	3/20^†^ (15)
Isolated	4/20^†^ (20)
Intrathoracic mass	1/24 (4)
Consolidation	11/24 (46)
Consolidation and miliary	5/24 (21)
Consolidation and nodules	2/24 (8)
Consolidation without nodules (isolated consolidation)	4/24 (17)
Unique consolidation	1/24 (4)
Association with ground-glass opacities	3/24 (13)
Central distribution	2/24 (8)
Diffuse distribution	1/24 (4)
Intrathoracic lymphadenopathies^‡^	14/24 (58)
Bilateral, hilar and symmetric	8/14 (57)
Non symmetric	6/14 (43)
Compressive lymphadenopathy	1/14 (7)
Necrotic lymphadenopathy	1/14 (7)
Atelectasis	1/24 (4)
Pleural effusion	3/24 (13)
Pericardial effusion	1/24 (4)

*Including one cluster sign; ^†^missing data; ^‡^intrathoracic lymphadenopathies were defined as a single abnormality, whether they were mediastinal, hilar, unilateral or bilateral. Axillary and cervical lymph nodes were not described on the thoracic imaging

### Diagnosis of the Pulmonary Focalization

Sixty patients (92%) had a bronchoscopy (Table 5). Among the 3 patients with a macroscopical lesion, one had a diffuse mucosal inflammation and interbronchial spurs thickening; one had an infiltrating mass of the right upper lobe with interlobular spurs enlargement and the last one had whitish secretions with areas of anthracosis.

**Table 5:** Laboratory findings in mycology and anatomical pathology and cytology by diagnostic procedures of proven HIV-associated pulmonary histoplasmosis cases

	No/total (%)
Bronchoscopy	60/65 (92)
Macroscopical abnormalities	3/60 (5)
BAL	59/60 (98)
Positive MDE	46/58 (79)
Positive culture*	40/54 (74)
Positive APC	7/13 (54)
Pulmonary biopsy	2/65 (3)
Positive APC	2/2 (100)
Tracheobronchial suctioning	2/65 (3)
Positive MDE	2/2 (100)
Positive culture	1/2 (50)
Sputum	8/65 (12)
Positive MDE	3/8 (38)
Positive culture	5/8 (63)

Patients may have several sampling sites. *on Sabouraud’s dextrose agar

*APC* anatomical pathology and cytology, *BAL* Broncho Alveolar Lavage; *MDE* mycological direct examination

PH cases were associated with an extrapulmonary detection of *H. capsulatum* in 78%, involving one, 2 or 3 other localizations in 42%, 25% and 12%, respectively. Bone marrow (27/33 patients), blood (16/23 patients), skin (9/14 patients) were the main detection sites associated. PH *stricto *sensu (without other localization) was found in 14/65 patients (22%), with 5/14 patients screened for histoplasmosis outside the pulmonary area. None of the 5 patients had a blood culture and one had a bone marrow sampling (detailed results in the supplementary appendix).

### Treatments and Outcome

All patients received an antifungal treatment, within the day of diagnosis suspicion in all patients but one.

Sixty-three patients (97%) were treated with induction doses (Table 6). One was started on treatment in the outpatient and one was started on maintenance therapy (itraconazole 400 mg/day) without induction.

**Table 6:** Treatments and outcomes of HIV-associated histoplasmosis pulmonary cases

	No/total (%) Median [Q1–Q3]
*Induction therapy*	63/65 (97)
Type of treatment	
Itraconazole 600 mg/day, po	43/63 (68)
Monotherapy	29/43 (67)
Failure of itraconazole and salvage therapy with liposomal amphotericin B*	1/43 (2)
Association with lipidic or deoxycholate formulation of amphotericin B	13/43 (30)
Median treatment duration (days) (n = 20)	6.5 [3–18.75]
Liposomal amphotericin B (L-AmB) 3–4 mg/kg/day, IV	20/63 (32)
Monotherapy	7/20 (35)
Salvage therapy after failure of itraconazole*	2/20 (10)
Association with itraconazole (with induction doses)	11/20 (55)
Median treatment duration (days) (n = 20)	9.5 [6–13.5]
Amphotericin B deoxycholate 1 mg/kg/day, IV	14/63 (22)
Monotherapy	12/14 (86)
Monotherapy followed by itraconazole (with induction doses)	2/14 (14)
Median treatment duration (days) (n = 6)	3 [3.5–15.25]
Time between diagnostic suspicion and treatment initiation	
≤ 1 day	62/63 (98)
> 1 day	1/63 (2)
*Maintenance therapy*	16/65 (25)
Itraconazole 400 mg/day, po	16/16 (100)
Voriconazole (no information about doses)^†^	1/16 (6)
Posaconazole (10 mL × 2/day, syrup and 400 mg × 2/day, po)	2/16 (13)
*Secondary prophylaxis*	
Itraconazole 200 mg/day, po	8/65 (12)
Overall antifungal treatment > 6 months	9/42 (21)
Overall antifungal treatment > 1 year	7/41 (17)
Outcome	
Death < 1 month after treatment initiation	14/65 (22)
Death > 1 month after treatment initiation	15/65 (23)
Lost to follow-up^‡^	14/65 (22)
Alive at the end of the study period	22/65 (34)
Meeting ≥ 1 severe case definition (WHO Guidelines 2020)	46/65 (71)
Respiratory^§^ or circulatory^II^ failure	11/26 (42)
Neurological signs	9/65 (14)
Renal failure^¶^	7/63 (11)
Coagulation anomalies**	29/59 (49)
Impaired WHO general performance score > 2	28/65 (43)

n = number of available data; *1 failure after 15 days of itraconazole 600 mg (induction doses), needing 9 days of L-AmB and 1 failure with itraconazole 400 mg (not represented in the itraconazole induction part), needing 13 days of L-AmB; †bad tolerance with a switch to posaconazole (previous intolerance to itraconazole); ‡missing medical appointment for more than one year; §clinical respiratory failure or oxygen blood pressure level < 70 mmHg; II mean blood pressure < 70 mmHg; ^¶^creatinine > 1.70 mg/dL; ^**^ platelet count < 150,000 cells/μL

Sixteen patients (25%) had a maintenance therapy. All were treated with itraconazole but 2 had vomiting or drug-drug interactions warranting a treatment switch to posaconazole and/or voriconazole. Nine patients completed antifungal therapy in 6 months and 7 patients in 12 months. Among the 49 patients without maintenance therapy, 26 deceased, 10 were lost to follow up, and 13 remained in care with an erratic compliance to antifungal therapy. The main reasons for treatment’s discontinuation were abscondence, noncompliance and death. Half of patients with maintenance therapy received a secondary prophylaxis. No histoplasmosis relapse was observed among the 22 patients alive and still in care by the end of the study period.

The case-fatality rate within one month after antifungal initiation was 22%. Seventy-one percent of patients met ≥ 1 WHO severe criteria with respiratory or circulatory failure, coagulation anomalies and impaired WHO performance status score > 2 in more than 40%.

## Discussion

We reported 65 cases of PH in PLHIV, including 22% of isolated pulmonary localization and 78% with dissemination. Considered as the AIDS-defining condition for 88% of cases, it was slightly over the 50–75% of DH cases described in PLHIV in the USA [1]. It may results from a high suspicion index among clinicians in our endemic area leading to systematic sampling and screening for histoplasmosis in patients with advanced HIV disease [18], rather than the clinical expression of South American *H. capsulatum* strains [19]. Still, patients under study were deeply immunocompromised with a median CD4 count of 24 cells/mm^3^, a concomitant AIDS-defining condition in one third, which is quite similar to previous reports on HIV-associated histoplasmosis [20–22].

Despite initiation of antifungal therapy, the case-fatality rate < 1 month was high (22%). Most of the deceased patients (64%) were included in the 1990’s when standards of care for both HIV and histoplasmosis where less effective. However, regardless of the time period, all but one presented with dyspnea and diffuse lung involvement on lung imaging, dyspnea being previously described as an independent prognostic factor associated with mortality [23].

Impaired WHO performance status score and fever were similar to the classical description of HIV-associated histoplasmosis [7, 9, 24]. Lymphadenopathies and hepatosplenomegaly showed differences when compared to previous reports of DH, less [9] and more [24, 25] common, respectively. No patient presented with chest pain, despite its classical description in non-HIV individuals [26], and only one patient had pericardial effusion on the CT-scan, which is concordant with pericarditis scarcity in HIV-associated histoplasmosis [1]*.* Hemoptysis, classically associated with tuberculosis, was reported in 3 patients. Forty-six patients met the 2020 WHO severity definition of DH, when 34 were treated with Amphotericin (liposomal or deoxycholate). This difference could be explained by the fact that the liposomal form was only available in the late 90’s and the deoxycholate form was avoided in patients with renal failure.

Associated to a suggestive clinical picture, medical imaging is the cornerstone for the diagnosis of PH but few data are available on PLHIV. We reported detailed data on both chest X-ray and CT-scan despite the low sensitivity of chest X-rays and the low number of CT-scan available in patients included before 2010.

Chest X-ray was the most commonly available examination and 13% were assessed as normal despite positive respiratory samples for *H. capsulatum*, which is similar to a subgroup of PLHIV with confirmed PH described previously [27]. An interstitial lung disease and a nodular pattern were mainly found, consistent with data reported in the literature [25, 27].

The main CT finding was miliary disease, consistent with the data of histoplasmosis in AIDS [28, 29]. About half of the miliary nodules were associated with consolidations, known to be the second most common presentation of DH [28, 30]. Three patients with ground-glass opacities probably had advanced disease, suggestive of heart failure, although data in medical reports were not consistent.

This presentation differs from that of immunocompetent patients, in whom histoplasmosis most often presents as solitary or few nodules, sometimes calcified [28, 31, 32]. In our immunocompromised patients, nodules, present in > 30% were the second most frequent abnormality, and were associated with miliary micronodules in one patient. Seven patients presented at least one excavated nodule or micronodule. In the literature, cavitary lesions have been described either in patients with acute nodular histoplasmosis or in case of chronic cavitary pulmonary histoplasmosis, occurring in patients with emphysema without intrathoracic lymphadenopathies [28, 30], while only 2 patients understudy had emphysema and all reported intrathoracic lymphadenopathies. One patient had a focal distribution of micronodules in the form of a “(sarcoid) cluster sign”, primarily described in thoracic sarcoidosis or tuberculosis with low bacilli rate [33, 34]. This patient had a history of tuberculosis of the right inferior lobe 6 years prior (without CT-scan at that time), whereas the micronodules were predominantly present in the left lung.

Intrathoracic lymphadenopathies were frequent, when considered rare [30] or not mentioned [28] in literature, and interestingly bilateral and symmetric, as usually described in sarcoidosis [35] but not in infectious diseases including tuberculosis. Only one patient presented with a necrotic lymphadenopathy, possibly related to his relative immunocompetence (295 CD4/mm^3^).

Unilateral distribution of PH usually occurs with a CD4 count > 300 cells/mm^3^ [1] but in our study the 2 patients with unilateral involvement had CD4 counts of 23 and 184 cells/mm^3^. We did not observed pulmonary abnormalities associated with chronic exposure (calcified nodules, histoplasmoma, broncholithiasis, or fibrosing mediastinitis) [28, 30].

Studies on HIV-associated histoplasmosis raise awareness on its non-specific clinical presentation, including the frequent miliary pattern on imaging, responsible for many misdiagnoses of tuberculosis and many subsequent preventable deaths [8, 28]. Yet, a recent review [36] on tomographic assessment of thoracic fungal disease in immunocompromised patients (including PLHIV) showed miliary pattern, consolidations, cavitations or thoracic lymphadenopathies as suggestive of histoplasmosis. It calls for more systematic screening for histoplasmosis and antifungal therapy initiation by physicians facing pulmonary diseases in advanced HIV patients.

Pulmonary co-infections between histoplasmosis and other pathogens are common in advanced HIV disease (PJP 15% [24]–25% [1], tuberculosis 2–26% [37], nontuberculous mycobacteria 17% [24], or CMV 20% [24]), even associated and not suspected pre-mortem [38], with major implications in the care and treatment outcome of histoplasmosis cases [37]. Nevertheless, when thinking of differential diagnoses, clinicians should keep in mind that histoplasmosis may be the main and even the only etiology of a miliary pattern in PLHIV [29, 39, 40].

Limitations of the present study were its retrospective design with potential selection biases and missing data. Including patients over a long time period, evolutions in the standard of care over time and exclusion of extrapulmonary proven histoplasmosis with abnormal chest imaging but no or negative respiratory sample may have resulted in selection and information biases.

However, to our knowledge, this study is the largest report with detailed insights into clinical and medical imaging findings on proven PH in PLHIV without concomitant pulmonary infections.

PH in PLHIV may occur at any time during the course of HIV infection, especially in advanced HIV patients. Differential diagnosis with tuberculosis or PJP may be challenging, notably in the context of coinfections. Waiting for the results of sampling procedures, antifungal therapy targeting *H. capsulatum* should be initiated early, notably when facing a miliary pattern. We call for updated algorithm on the diagnosis and management of pulmonary diseases in PLHIV, where histoplasmosis would be systematically evocated, screened and treated regardless of the physicians’ settings.

### Supplementary Information

Below is the link to the electronic supplementary material.

Supplementary file1 (DOCX 13 kb)
