# Supplementary material for: Pulmonary Histoplasmosis in People Living with Human Immunodeficiency Virus in French Guiana: Clinical Epidemiology, Medical Imaging and Prognostic
Source: Mycopathologia. 2023 Oct 15;188(6):1065–78. doi: 10.1007/s11046-023-00799-x (PMC10687118; doi:10.1007/s11046-023-00799-x)
Supplement: Supplementary file 1 — Supplementary file1 (DOCX 13 kb) [file 11046_2023_799_MOESM1_ESM.docx]

### Supplementary appendix

### Extrapulmonary histoplasmosis

|  | No/Total (%) |
| --- | --- |
| **Isolated pulmonary histoplasmosis** | 14/65 (22) |
| No other infected organ and at least one other site screened | 5/65 (8) |
| **Disseminated histoplasmosis** | 51/65 (78) |
| 1 other infected organ | 27/65 (42) |
| 2 other infected organs | 16/65 (25) |
| 3 other infected organs | 8/65 (12) |
| **Extrapulmonary infected sites** |  |
| Bone marrow | 27/33 (82) |
| Blood | 16/23 (70) |
| Mucocutaneous | 9/14 (64) |
| Liver | 8/8 (100) |
| Lymphadenopathy | 7/7 (100) |
| Lower digestive tract | 7/9 (78) |
| Urine | 4/10 (40) |
| Cerebrospinal Fluid | 2/10 (20) |
| Upper digestive tract | 1/7 (14) |
| Peritoneal^*^ | 1/1 (100) |

*no other digestive organs biopsied.
